# Immune checkpoint-targeted antibodies: a room for dose and schedule optimization?

**DOI:** 10.1186/s13045-021-01182-3

**Published:** 2022-01-15

**Authors:** Christophe Maritaz, Sophie Broutin, Nathalie Chaput, Aurélien Marabelle, Angelo Paci

**Affiliations:** 1 Pharmacology Department, U1030 INSERM, University Paris-Saclay, Gustave Roussy Cancer Campus, Villejuif, France; 2 Laboratory for Immunomonitoring in Oncology (LIO), Faculty of Pharmacy, University Paris-Saclay, Gustave Roussy Cancer Campus, Villejuif, France; 3 Drug Development Unit (DITEP), LRTI U1015 INSERM, Gustave Roussy, Villejuif, France; 4 Pharmacokinetic Unit, Faculty of Pharmacy, University Paris-Saclay, Chatenay-Malabry, France

**Keywords:** Immunotherapy, Therapeutic antibodies, Dosing interval, Pharmacokinetics, Oncology

## Abstract

Anti-CTLA-4 and anti-PD-1/PD-L1 immune checkpoint inhibitors are therapeutic monoclonal antibodies that do not target cancer cells but are designed to reactivate or promote antitumor immunity. Dosing and scheduling of these biologics were established according to conventional drug development models, even though the determination of a maximum tolerated dose in the clinic could only be defined for anti-CTLA-4. Given the pharmacology of these monoclonal antibodies, their high interpatient pharmacokinetic variability, the actual clinical benefit as monotherapy that is observed only in a specific subset of patients, and the substantial cost of these treatments, a number of questions arise regarding the selected dose and the dosing interval. This review aims to outline the development of these immunotherapies and considers optimization options that could be used in clinical practice.

## Introduction

**Fig. 1:**
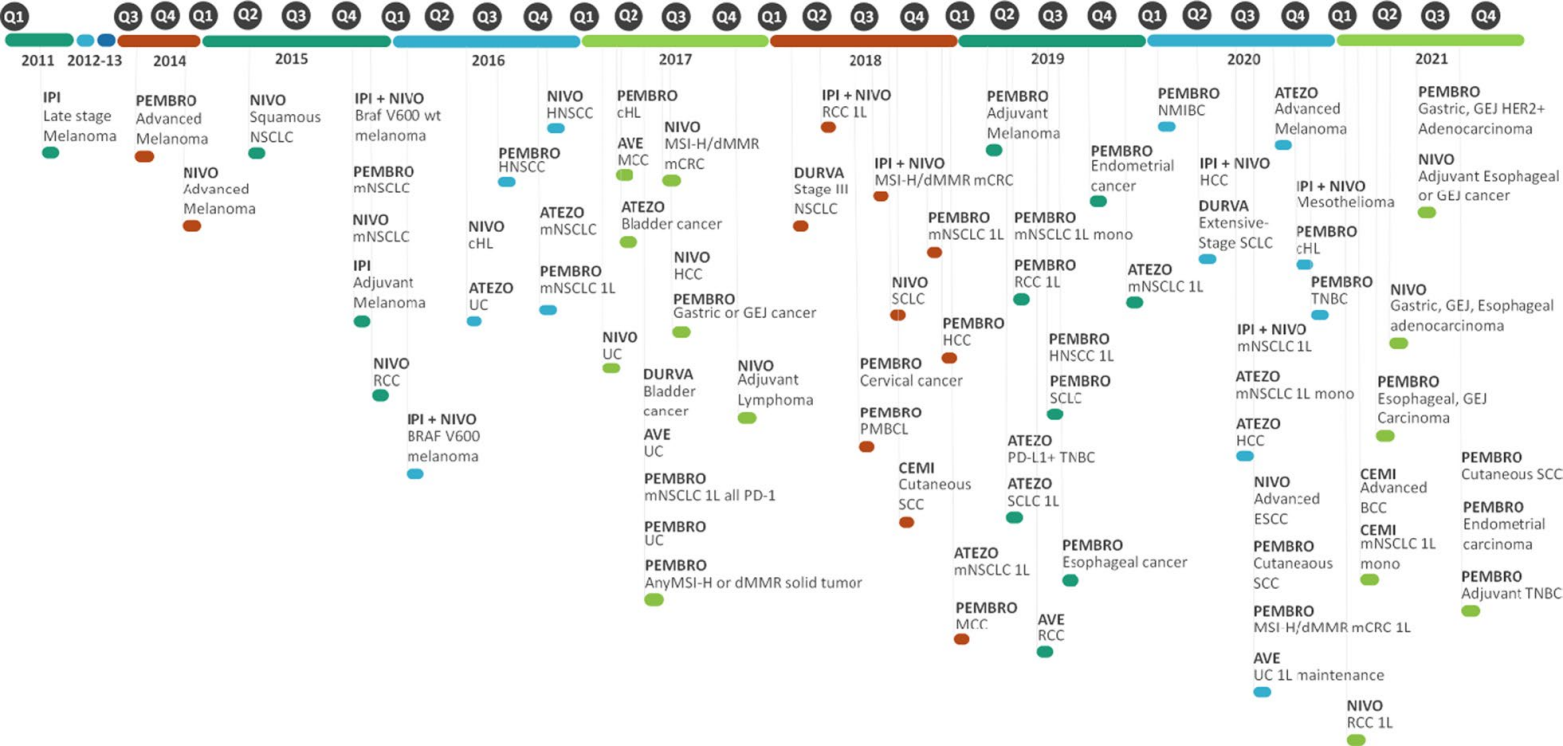
Timeline of approved ICIs. IPI, ipilimumab; NIVO, nivolumab; PEMBRO, pembrolizumab; ATEZO, atezolizumab; AVE, avelumab; DURVA, durvalumab; CEMI, cemiplimab. mNCSLC, metastatic non-small cell lung cancer; wt, wild type; RCC, renal cell carcinoma; cHL, classical Hodgkin lymphoma; UC, urothelial cancer; HNSCC, head and neck squamous cell carcinoma; MCC, Merkel cell carcinoma; mCRC, metastatic colorectal cancer; HCC, hepatocarcinoma; GEJ, gastroesophageal junction; PMBCL, primary mediastinal B-cell lymphoma; SCLC, small cell lung cancer; SCC, squamous cell carcinoma; TNBC, triple negative breast cancer; NMIBC, non-muscle-invasive bladder cancer; ESCC, esophageal squamous cell carcinoma; BCC, basal cell carcinoma

Therapeutic antibodies have significantly improved the management of cancer. The first therapeutic antibodies used in oncology directly targeted cancer cells to promote their destruction. Increased knowledge has led to a better understanding of the tumor environment. An important step has been the identification of certain tumor cells able to disrupt the immune response through the expression of molecules called immune checkpoints. Targeting these molecules with specific engineered antibodies called immune checkpoint inhibitors (ICIs) has greatly improved clinical outcomes. ICIs reinvigorate immunity. The first available ICI targeted cytotoxic T lymphocyte antigen-4 (CTLA-4). Since then, other ICIs targeting the PD-1 receptor (programmed cell death protein-1) or its ligand PD-L1 have been added to the clinical armamentarium. New indications have been extended to several cancer types (Fig. 1).

While limited to a specific subset of patients, the efficacy of ICIs is compelling since they have improved clinical outcomes for several cancer types, leading to their approval in multiple solid and hematologic malignancies [1]. Their interest remains and many clinical trials are underway. Initially, doses were based on body weight at regular intervals, comparable to those used for chemotherapy (usually every 2 or 3 weeks), as body weight was thought to be a major factor in interpatient pharmacokinetic variability [2]. Currently, the dosing is directed toward the administration of fixed doses at regular intervals, which tend to lengthen, every 4 or 6 weeks. Fixed dosing is particularly suitable for compounds with a wide therapeutic range, which appears to be the case for ICIs, and has the advantage of facilitating preparation and reducing drug waste [2]. Although fixed dosing is associated with a substantial weight-dependent change in plasma concentrations, particularly for extreme body weights, since the minimum effective dose appears to be largely exceeded, it is assumed that the impact on treatment outcome is negligible [3, 4]. The route of administration, exclusively by intravenous infusion, is also directed toward a formulation allowing subcutaneous injection, as well as intratumoral delivery. Clinical studies of currently available ICIs (except for ipilimumab) have not identified dose-limiting toxicity or dose-related efficacy, which tends to use doses that are well above the minimum effective doses. In addition, several studies have described exposure–response relationships where concentration alone does not appear to be sufficient, but determining the clearance of these ICIs that incorporates different covariates appears promising [4–6]. Currently available ICIs are immunoglobulins of isotype G (IgG), exhibiting the same pharmacokinetic properties. Their volume of distribution is close to plasma volume, with a limited tissue distribution and target-antigen binding affinity playing an important role. Metabolism and elimination do not involve renal and hepatic functions, due to their high molecular weight and absence of CYP enzyme involvement. Metabolism and elimination occur through both specific (target-mediated, fast) and non-specific (FcRn-mediated, slow) routes, resulting in nonlinear and linear elimination, respectively. At current doses, saturation of the target-mediated route occurs rapidly, with the non-specific route becoming predominant, accounting for the long half-life of these compounds and a slow clearance [3, 5]. Given these pharmacokinetics, the high cost of these treatments, and the clinical benefit as monotherapy observed only in a subset of patients, it may be appropriate to rethink doses, dosing intervals, and treatment durations of these therapeutic antibodies to optimize their clinical use. The dose trends and indications for these ICIs are compiled in Table 1. Efficacy and safety end points by dose and indication that are discussed below are summarized in Table 2.

**Table 1:** Indications and dosages of ICIs

Target	Drug	Half-life	Regimen	Indication	First approved doses	Current approved doses
CTLA-4	Ipilimumab	~15 days	Monotherapy	Advanced melanoma	3 mg/kg Q3W total of 4 doses	
			Monotherapy	Adjuvant melanoma*	10 mg/kg Q3W for 4 doses followed by 10 mg/kg Q12W up to 3 years	
			In combination with nivolumab	Advanced melanoma HCC*	3 mg/kg Q3W total of 4 doses	
				Advanced RCC MSI-H/dMMr mCRC	1 mg/kg Q3W total of 4 doses	
				mNSCLC* mesothelioma	1 mg/kg Q6W up to 2 years	
			In combination with nivolumab and chemotherapy	mNSCLC	1 mg/kg Q6W up to 2 years	
PD-1	Nivolumab	~25 days	Monotherapy	Advanced melanoma mNSCLC Advanced RCC Hodgkin lymphoma HNSCC Urothelial carcinoma HCC* Esophageal cancer MSI-H/dMMr mCRC*	3 mg/kg Q2W	240 mg Q2W or 480 mg Q4W^†^
				Adjuvant melanoma Adjuvant Esophageal or GEJ cancer		240 mg Q2W or 480 mg Q4W^†^ up to 1 year
				MSI-H/dMMr mCRC (< 40 kg)*		3 mg/kg Q2W
			In combination with ipilimumab	Advanced melanoma HCC*	1 mg/kg Q3W for 4 doses followed by 3 mg/kg Q2W	1 mg/kg Q3W for 4 doses followed by 240 mg Q2W or 480 mg Q4W
				mNSCLC*	3 mg/kg Q2W up to 2 years	
				Mesothelioma	3 mg/kg Q2W	360 mg Q3W up to 2 years
				Advanced RCC MSI-H/dMMr mCRC	3 mg/kg Q3W for 4 doses followed by 3 mg/kg Q2W	3 mg/kg Q3W for 4 doses followed by 240 mg Q2W or 480 mg Q4W
				MSI-H/dMMr mCRC (< 40 kg)	3 mg/kg Q2W	
			In combination with ipilimumab and chemotherapy	mNSCLC	360 mg Q3W up to 2 years	
			In combination with cabozantinib	Advanced RCC	240 mg Q2W up to 2 years	240 mg Q2W or 480 mg Q4W up to 2 years
			In combination with chemotherapy	Gastric, GEJ, Esophageal cancer*	240 mg Q2W or 360 mg Q3W up to 2 years	
	Pembrolizumab	~25 days	Monotherapy	Advanced melanoma mNSCLC mSCLC* Hodgkin lymphoma Mediastinal B-cell lymphoma* HNSCC Urothelial carcinoma HCC* Gastric, GEJ, Esophageal cancer* Cervical cancer* Merkel cell carcinoma* MSI-H/dMMr mCRC High-risk NMIBC* Cutaneous SCC*	2 mg/kg Q3W up to 2 years	200 mg Q3W or 400 mg Q6W up to 2 years
				Adjuvant melanoma	200 mg Q3W up to 1 year	200 mg Q3W or 400 mg Q6W up to 1 year
				Pediatrics	2 mg/kg Q3W up to 2 years	
			In combination with chemotherapy	mNSCLC HNSCC Esophageal cancer Advanced TNBC*	2 mg/kg Q3W up to 2 years	200 mg Q3W or 400 mg Q6W up to 2 years
			In combination with trastuzumab and chemotherapy	Gastric, GEJ cancer*		
			In combination with axitinib	Advanced RCC		
			In combination with lenvatinib	Endometrial carcinoma*		
	Cemiplimab	~19 days	Monotherapy	Cutaneous SCC BCC mNSCLC	3 mg/kg Q2W	350 mg Q3W
PD-L1	Avelumab	~4 days	Monotherapy	Metastatic Merkel cell carcinoma Urothelial cancer	10 mg/kg Q2W	800 mg Q2W
			In combination with axitinib	Advanced RCC		
	Atezolizumab	~27 days	Monotherapy	Urothelial cancer mNSCLC	1200 mg Q3W	840 mg Q2W or 1200 mg Q3W or 1680 mg Q4W
			In combination with nab-paclitaxel	Advanced TNBC	840 mg Q2W	
			In combination with bevacizumab and chemotherapy	mNSCLC	1200 mg Q3W	840 mg Q2W* or 1200 mg Q3W or 1680 mg Q4W*
			In combination with chemotherapy	mSCLC		
			In combination with bevacizumab	HCC		
			In combination with cobimetinib and vemurafenib	Advanced melanoma*		
	Durvalumab	~18 days	Monotherapy	Unresectable NSCLC	10 mg/kg Q2W up to 1 year	10 mg/kg Q2W or 1500 mg Q4W up to 1 year
				Patients < 30 kg	10 mg/kg Q2W up to 1 year	
			In combination with chemotherapy	Extensive-stage SCLC	1500 mg Q3W for 4 doses followed by 1500 mg Q4W	1500 mg Q3W for 4 doses followed by 1500 mg Q4W
				Patients < 30 kg	20 mg/kg Q3W for 4 doses followed by 10 mg/kg Q2W	

HCC, hepatocarcinoma; RCC, renal cell carcinoma; mCRC, metastatic colorectal cancer; mNCSLC, metastatic non-small cell lung cancer; HNSCC, head and neck squamous cell carcinoma; GEJ, gastroesophageal junction; mSCLC, metastatic small cell lung cancer; NMIBC, non-muscle-invasive bladder cancer; SCC, squamous cell carcinoma; TNBC, triple negative breast cancer; BCC, basal cell carcinoma

*Only FDA approved

^†^480 mg only approved by EMA for Melanoma, RCC (advanced and adjuvant) and Esophageal or GEJ cancer (adjuvant)

**Table 2:** Summary of efficacy and safety end points for ICIs by indication and dosage observed in development studies

Target	Drug	Indication	Dose	ORR (%)	PFS (% at 24 weeks; otherwise median)	OS (% at 1 year; otherwise median or specified)	G3-G4 toxicity (%)
CTLA-4	Ipilimumab [7–11]	Melanoma	3 mg/kg Q3W	[4.2–19]	12.9% [2.63–2.9] months	[39.3–60.9]%	[15–27.3]
			10 mg/kg Q3W	[11.1–15]	18.9% [2.56–2.8] months	[44.2–54.3]%	[31–34]
			0.3 mg/kg Q3W	0	2.7%	39.6%	
		RCC	1 mg/kg Q3W	40.4	55.6%	81%	38.3
			3 mg/kg Q3W	40.4	63.8%	85%	61.7
PD-1	Nivolumab [9, 12–19]	Melanoma	0.1 mg/kg Q2W	[29–35]	[40–41]%		[0–5]
			0.3 mg/kg Q2W	[19–28]	[31–35]%		[0–3]
			1 mg/kg Q2W	[30 31]	[45–51]%		[6–12]
			3 mg/kg Q2W	[31–43.7]	[39–55]% 6.9 months	72.9%	[4–16.3]
			10 mg/kg Q2W	[9–20]	[30–45]%		[8–21]
		NSCLC	1 mg/kg Q2W	[3–6]	[26–46]%	33%	15.2
			3 mg/kg Q2W	[24–32]	[40–41]%	56%	13.5
			10 mg/kg Q2W	[18–20]	[24–33]%	38%	13.6
		RCC	0.3 mg/kg Q2W	20	30%	18.2%	5
			1 mg/kg Q2W	[24–28]	[47–50]%		12
			2 mg/kg Q2W	22	30%	25.5%	17
			10 mg/kg Q2W	31	67%	24.7%	13
		Mixed	480 mg Q4W				14.8
	Pembrolizumab [20–31]	Melanoma	2 mg/kg Q3W	[21–32.9]	45%	58%	[8–15]
			10 mg/kg Q2W	[33.7–52]			[13.3–22.8]
			10 mg/kg Q3W	[26–35.9]	37%	53%	[3.6–16]
		NSCLC	2 mg/kg Q3W	[15–25]	3.9 months	10.4 months	13
			10 mg/kg Q2W	[19.3–21]			9
			10 mg/kg Q3W	[19.2–25]	4 months	12.7 months	[3.6–16]
		Hodgkin lymphoma	10 mg/kg Q2W	65	69%	100% at 24 weeks	22
			200 mg Q3W	69			5.4
		HNSCC	10 mg/kg Q2W	18			23
			200 mg Q3W	16	23%	59% at 24 weeks	15
		Urothelial cancer	10 mg/kg Q2W	26	2 months	13 months	15
			200 mg Q3W	24	2.1 months	10.3 months	15
	Cemiplimab [32]	Cutaneaous SCC	350 mg Q3W	41	10.4 months	76.1%	39.3
			3 mg/kg Q2W	49	18.4 months	81.3%	50.8
PD-L1	Avelumab [33–36]	RCC	10 mg/kg Q2W	[51.4–58]	13.8 months		[58–71.2]
		Merkel cell carcinoma	10 mg/kg Q2W	31.8	40%	69% at 24 weeks	5
		Mixed	1 mg/kg Q2W	0			25
			3 mg/kg Q2W	0			0
			10 mg/kg Q2W	13			33
			20 mg/kg Q2W	10			14
	Atezolizumab [37, 38]	NSCLC	1200 mg Q3W	14	2.8 months	13.8 months	13
			1; 3; 10; 15, 20 mg/kg Q3W	23	45%		
		Melanoma		30	41%		
		RCC		14	48%		
		Urothelial cancer	1200 mg Q3W	15	2.1 months	7.9 months	16
	Durvalumab [39–42]	SCLC	1500 mg Q3W	68	45%	54%	22
		Urothelial cancer	10 mg/kg Q2W	31			5
		NSCLC	3 mg/kg Q2W	0			0
			10 mg/kg Q2W	33			67
			15 mg/kg Q2W	33			72
			20 mg/kg Q2W	50			61
			10 mg/kg Q2W	0	17.2 months	83.1%	[30.5–53]

ORR, Objective response rate; PFS, Progression-free survival; OS, Overall survival; G3–G4, grade3–grade4

## Therapeutic antibody targeting CTLA-4

### Ipilimumab, human IgG1

#### Clinical development

##### Melanoma

In a Phase I/II study with interleukin-2, patients received ipilimumab at the dose of 0.1, 0.3, 1, 2 or 3 mg/kg every 3 weeks (Q3W) for a total of 3 doses. An objective response rate (ORR) of 33% was found in the 0.1–2 mg/kg cohorts and 21% in the 3 mg/kg cohort [43]. In another study combining ipilimumab 3 mg/kg with peptides from the gp100 melanoma-associated antigen, patients received ipilimumab 3 mg/kg Q3W or an initial dose of 3 mg/kg followed by a dose of 1 mg/kg Q3W. The ORR was similar between the two regimens (12.5%), and there were no significant differences in toxicity. Autoimmune manifestations were reversible and appeared to be associated with clinical response [44]. In the same study, new patients were added to evaluate dose escalation schedule without vaccination. Patients received an initial dose of ipilimumab of 3 mg/kg or 5 mg/kg Q3W and increased to 5 and then 9 mg/kg. The study concluded in an increase in grade III/IV autoimmune manifestations related to the dose with no improvement in clinical response [45]. To determine the optimal dose of ipilimumab, a Phase II study evaluated ipilimumab administered at 10 mg/kg, 3 mg/kg and 0.3 mg/kg Q3W, for a total of 4 doses, then Q12W. The results indicated a clear dose-dependent effect, with higher doses of ipilimumab associated with higher ORR but also increased toxicity [7]. A Phase III study was conducted to confirm the benefit/risk profile of ipilimumab at 3 versus 10 mg/kg Q3W. Although administration of ipilimumab at a higher dose was associated with significantly longer overall survival OS (15.7 months vs. 11.5 months; *p* = 0.04), treatment-related serious adverse events were also significantly increased. High doses of ipilimumab are therefore not recommended [8]. In the Phase II CheckMate 069 trial, nivolumab 1 mg/kg plus ipilimumab 3 mg/kg versus ipilimumab alone showed a significantly greater ORR and progression-free survival (PFS) [46]. In the Phase III CheckMate 067 trial, nivolumab plus ipilimumab versus ipilimumab alone significantly improved PFS (11.5 months vs. 2.9 months; *p* < 0.001) with a higher incidence of grade III/IV treatment-related adverse events (55% vs. 27.3%) [9]. Neither the dose nor the dosing interval have been further evaluated.

##### Renal cell carcinoma

The Phase I CheckMate 016 trial evaluated the combination nivolumab plus ipilimumab. In two arms of this study, patients received ipilimumab 1 mg/kg plus nivolumab 3 mg/kg Q3W or ipilimumab 3 mg/kg plus nivolumab 1 mg/kg Q3W for a total of 4 doses. In the arm receiving the combination with ipilimumab 3 mg/kg, the occurrence of grade III–IV adverse events was greater (61.7% vs. 38.3%) [10]. These observations guided the choice of the dose of 1 mg/kg for ipilimumab in the pivotal Phase III CheckMate 214 trial [47].

#### Potential dose and schedule optimization for ipilimumab

It is interesting to note the differences between the doses for melanoma compared with renal cell carcinoma. Current data do not allow for a head-to-head comparison of these two doses, nor do they allow for a conclusion on an optimal dosing interval. To this end, the results of the Phase II PRISM study, which began in August 2016, are expected by December 2021. The objective of this study is to determine whether extending the dosing interval of ipilimumab (1 mg/kg Q3W or Q12W for a total of 4 doses) in combination with nivolumab will reduce toxicity without compromising the efficacy in the first-line treatment of renal cell carcinoma. The results could impact the clinical practice in renal cell carcinoma in addition to the current regimen in melanoma [48].

Ipilimumab had comparable efficacy in doses ranging from 3 to 10 mg/kg with a lower toxicity at 3 mg/kg [11]. The saturation of the target receptor determined in vitro at 20 µg/mL was almost total for doses equivalent to 3 mg/kg Q3W [7]. A clear exposure–response relationship was found between steady-state trough levels (C_min,ss_) and OS at doses ranging from 0.3 to 10 mg/kg. Patients in the highest quartile of C_min,ss_ (82.1 µg/mL) had an approximatively fourfold increase in OS (24.3 months) compared to patients in the lowest quartile, i.e., below the target saturation threshold (8.52 µg/mL; 6.51 months) [49]. High interpatient variability has been reported for ipilimumab. Clearance does not appear to be time-dependent but rather is influenced by the patient’s weight at the start of treatment, supporting the use of a weight-based dose rather than a fixed dose [6]. Given the significant interpatient variability, the exposure–response relationship and the dose-dependent toxicity, the implementation of therapeutic monitoring for ipilimumab seems relevant, especially when combined with nivolumab.

## Therapeutic antibodies targeting PD-1

### Nivolumab, human IgG4

#### Clinical development

In the first Phase I multi-tumor study, a total of 39 patients received nivolumab at 0.3, 1, 3 or 10 mg/kg on a weekly basis for 8 weeks. Nivolumab showed a favorable safety profile with evidence of antitumor activity [50].

##### Melanoma

Nivolumab monotherapy was initially evaluated in a Phase I study with or without a polypeptide vaccine. In this study, 90 patients received nivolumab 1, 3 or 10 mg/kg Q2W for 24 weeks and then Q12W for 2 years. Two cohorts of 10 and 11 patients received nivolumab 1 mg/kg and 10 mg/kg, respectively, while 79 patients in four distinct cohorts received nivolumab 3 mg/kg. The ORR was equivalent between patients receiving 1 or 3 mg/kg. The authors concluded that nivolumab showed promising antitumor activity associated with a good safety profile at 3 mg/kg [12]. A subgroup analysis with doses ranging from 0.1 to 10 mg/kg Q2W for 96 weeks had comparable efficacy and safety profile regardless of the dose [13]. The maximum tolerated dose (MTD) was not reached, and the dose of 3 mg/kg Q2W was selected [14, 51]. The dosing has since evolved to a fixed dose of 240 mg Q2W or 480 mg Q4W. The dose and dosing interval used for development in combination with ipilimumab have already been discussed above.

##### Other indications

In a Phase II renal cell carcinoma study, patients had comparable efficacy and safety of nivolumab monotherapy at doses of 0.3, 2 and 10 mg/kg Q3W [15]. The authors explained the choice of 3 mg/kg Q2W on the results of a Phase I study where this dosing has not been described [13, 52]. In the Phase III CheckMate-025 trial, nivolumab 3 mg/kg Q2W was compared to everolimus. The OS was significantly prolonged with nivolumab (HR: 0.75; *p* = 0.002) and the safety profile favored nivolumab [53]. The potential of nivolumab + ipilimumab combination was evaluated in the Phase I CheckMate-016 dose escalation study as presented above, using the 3 mg/kg nivolumab + 1 mg/kg ipilimumab Q3W regimen, due to the toxicity of ipilimumab at higher doses [10]. An analysis from 129 NSCLC patients (54 squamous/74 non-squamous) receiving nivolumab 1, 3 or 10 mg/kg Q2W for 96 weeks showed a greater clinical activity in the 3 mg/kg cohort [16]. As this 3 mg/kg Q2W dose was associated with antitumor activity and an acceptable safety profile in all indications, no further dose exploration was conducted, and the dosing evolved to the fixed dose [54–58].

#### Potential dose and schedule optimization for nivolumab

In the initial clinical program of nivolumab suppress at with doses ranging from 0.1 to 10 mg/kg, the ORR was equivalent regardless of the dose in the treatment of melanoma and renal cell carcinoma. It was higher at 3 and 10 mg/kg in NSCLC [17]. These considerations supported the choice of the 3 mg/kg dose in monotherapy for all indications. The dosing has evolved to a fixed dose, based on an in silico analysis from clinical data of 90 patients with advanced tumors (melanoma, NSCLC and renal cell carcinoma) showing a comparable benefit-risk profile of nivolumab 240 mg Q2W versus 3 mg/kg [59]. A second in silico analysis showed an equivalent pharmacokinetic and safety profile between 240 mg Q2W and 480 mg Q4W fixed doses in 3817 patients with solid tumors (melanoma, NSCLC and renal cell carcinoma), leading to the approval of the 480 mg Q4W fixed dose [18]. From a pharmacokinetic standpoint, it appears that administration of the 480 mg Q4W fixed dose results in a higher Cmax and lower Cmin at steady state, comparable to 3 mg/kg or 240 mg Q2W doses depending on the indication [60]. The 240 mg Q2W fixed dose corresponds to a standardization on a 80 kg patient receiving 3 mg/kg. Safety and efficacy data for 480 mg fixed dose for low-weight patients are rare. Some countries have adopted an alternative dosing strategy at 6 mg/kg Q4W for patients with body weight below 80 kg while keeping approved fixed dose at 480 mg Q4W for patients of 80 kg and more in order to limit overexposure and costs [61].

The optimal duration of nivolumab treatment also remains an open question. An exploratory analysis of the CheckMate-153 trial in NSCLC showed an OS and PFS benefit of continuous administration compared to a 1-year treatment duration [62]. A retrospective French real-world study analyzed the duration of nivolumab treatment in 65 long-term survivors with NSCLC and tends to show a potential benefit of a treatment that last more than 2 years [63]. Another real-world retrospective study analyzed outcomes of patients with NSCLC who discontinued nivolumab for reasons other than disease progression. Of the 17 patients, 6 had a particularly long PFS (> 6 months), 5 of which had discontinued nivolumab due to immunity-related adverse events [64]. The occurrence of such adverse events is thought to be related to a deep reactivation of antitumor immunity, initiated by nivolumab, which would persist over time after treatment discontinuation.

Saturation of target receptors is achieved with doses ≥ 0.3 mg/kg [65]. interpatient variability in nivolumab clearance of approximately 30% is reported, particularly depending on the patient’s weight and disease stage [66, 67]. In melanoma, nivolumab clearance at baseline was shown to be a significant predictive factor of OS [68]. Clearance decreases over time, which may be due to disease evolution. Patients with a high tumor burden have a faster nivolumab clearance from the start of the treatment, and in responders, nivolumab clearance is slower over time with decreasing tumor burden [67]. Determining the trough serum concentration of nivolumab after the first administration (2–4 weeks later) could be a tool to assess clearance and thus identify patients who will not benefit from treatment (fast clearance). For those patients, a higher dose level could be an option but has not been evaluated so far. For responders, monitoring of the nivolumab serum concentration may allow adjustment of the dosing interval over time based on the clearance. Given that nivolumab has a half-life of 25 days, the use of pharmacological monitoring assessing serum trough concentrations could show that for a patient, 480 mg Q8W or more is sufficient, which would represent a substantial saving in terms of cost, as well as hospital time for preparation and administration. It could also have an impact on tolerance. Patients would rather come to the hospital less often, knowing that their exposure to the treatment is adequate [69].

### Pembrolizumab, humanized IgG4

#### Clinical development

##### Melanoma

In the Phase I KEYNOTE-001 study, 135 patients received pembrolizumab 10 mg/kg Q2W or Q3W or 2 mg/kg Q3W. Higher incidence of adverse events was observed for patients receiving the highest dose. The ORR ranged from 25% in the 2 mg/kg Q3W cohort to 52% in the 10 mg/kg Q2W cohort, suggesting a dose–response effect that was not subsequently replicated [20]. Pembrolizumab 2 versus 10 mg/kg Q3W was then compared in 173 patients previously treated with ipilimumab or MEK / BRAF inhibitors. Unlike the first analysis, no difference in efficacy or safety was reported between the two doses [21]. The Phase II KEYNOTE-002 trial evaluated pembrolizumab 2 versus 10 mg/kg Q3W. Regardless of the dose, pembrolizumab significantly prolonged PFS versus chemotherapy (*p* < 0.0001) [70]. In the phase III KEYNOTE-006 trial evaluating pembrolizumab 10 mg/kg Q2W or Q3W, the efficacy was comparable between the two [22]. In the adjuvant setting, the phase III KEYNOTE-054 trial evaluating the fixed dose of 200 mg Q3W showed a significant longer recurrence-free survival with pembrolizumab versus placebo (*p* < 0.001) [71].

##### Non-small cell lung cancer

In the Phase I KEYNOTE-001 study, 495 patients received pembrolizumab 2 or 10 mg/kg Q3W or 10 mg/kg Q2W. The safety profile and efficacy were comparable across the different treatment groups. The Phase II/III KEYNOTE-010 trial assessed pembrolizumab 2 or 10 mg/kg Q3W. Efficacy and safety were comparable between the two doses, so the authors encouraged the choice of the lowest dose 2 mg/kg Q3W [23]. Clinical development continued with the Phase III KEYNOTE-024 trial with a fixed dose of 200 mg Q3W [72]. The fixed-dose indication was extended to the squamous NSCLC with the positive results of the Phase III KEYNOTE-407 trial [73], to the non-squamous NSCLC in combination with chemotherapy with the results of the Phase III KEYNOTE-189 [74], and to the first-line treatment of metastatic NSCLC in monotherapy with the results of the Phase III KEYNOTE-042 [75].

##### Renal cell carcinoma

In a Phase Ib dose-finding study, the safety and efficacy of axitinib in combination with pembrolizumab 2 mg/kg Q3W was evaluated. Among the 51 patients treated, 73% achieved an objective response [76]. These results were confirmed by the phase III KEYNOTE-426 trial of pembrolizumab at 200 mg Q3W in combination with axitinib [77].

##### Other indications

In KEYNOTE trials for urothelial carcinoma, head and neck squamous cell carcinoma, and classical Hodgkin lymphoma, the regimen used was initially 10 mg/kg Q2W with a promising antitumor efficacy associated with a favorable safety profile. The positive results were confirmed for the pembrolizumab dose of 200 mg Q3W [24–29, 78, 79].

#### Potential dose and schedule optimization for pembrolizumab

Clinical development of pembrolizumab began with a weight-based dose (2 mg/kg Q3W) to evolve to the fixed dose of 200 mg Q3W. Pembrolizumab had a favorable safety profile at all doses evaluated, ranging from 0.005 to 10 mg/kg, and no MTD was reached [80]. Pharmacokinetic data showed an equivalent exposure between the 2 mg/kg Q3W and 200 mg Q3W doses in the treatment of solid tumors, mainly melanoma and NSCLC. This corresponds to a standardization on a 100 kg patient. For weight-based dosing, low-weight patients tend to have twice as low exposure as high-weight patients, and this trend is reversed when switching to fixed doses, low-weight patients have twice as much exposure as high-weight patients. In general, switching to the fixed dose of 200 mg results in greater exposure than when using the weight-based dose, regardless of patient weight. Since all these variations remain within the window defined as therapeutic, based on the efficacy and safety data from previous clinical studies, it was concluded that the different doses are equivalent. However, based on this modeling analyses, the fixed dose of 154 mg Q3W had the most comparable pharmacokinetic profile to the 2 mg/kg dose [81]. The 200 mg Q3W fixed dose was selected, which leads to an increased dose of 23%, with no impact on efficacy and safety, but representing a significant additional cost. The 200 mg Q3W fixed dose then evolved to 400 mg Q6W based on an in silico pharmacokinetic modeling study demonstrating exposure equivalence between 400 mg Q6W, 2 mg/kg Q3W and 200 mg Q3W [82].

Interestingly, there are data suggesting a long-term benefit of pembrolizumab after discontinuation of treatment. This was observed in the long-term follow-up of the KEYNOTE-001 study in melanoma patients. Comparable disease-free survival rates at 24 months were reported in patients with a complete response who did or did not continue pembrolizumab. In addition, 63.8% of patients who discontinued pembrolizumab after achieving a complete response did not receive subsequent cancer therapy [83]. The underlying mechanism is not known but suggests the induction and persistence of a long-lasting memory T cell response.

The pharmacokinetic data of pembrolizumab showed a linear time-dependent clearance profile for doses ranging from 0.3 to 10 mg/kg [80, 84, 85]. The profile is nonlinear for doses below 0.3 mg/kg [80, 85]. A PD-1 receptor saturation rate greater than 95% is achieved for doses higher than 1 mg/kg [80]. Clinical data confirm that there is no exposure–response relationship for doses ranging from 2 to 10 mg/kg in melanoma [21, 30, 70], NSCLC [23], and other solid tumors [80]. As with nivolumab, higher clearance of pembrolizumab has been linked to disease severity and OS [86, 87]. The efficacy of lower doses and longer dosing intervals has not been explored.

### Cemiplimab, human IgG4

#### Clinical development

##### Cutaneaous squamous cell carcinoma

Clinical development was rapid with a 26-patient Phase I study evaluating doses ranging from 1 to 10 mg/kg Q2W over 48 weeks. One patient who received cemiplimab 10 mg/kg Q2W achieved a durable complete response (more than 16 months) even after treatment discontinuation [88]. As with pembrolizumab and nivolumab, the dose/efficacy curve is a plateau for all doses evaluated [89]. The dose of 3 mg/kg Q2W for 54 weeks, with a possible extension to 96 weeks has been selected for the Phase II. The reasons were not specified. This Phase II trial was amended to include a group of 56 patients receiving a fixed dose of 350 mg Q3W for 96 weeks, showing equivalent efficacy to the weight-based dose [32].

#### Potential dose and schedule optimization for cemiplimab

Evolution to the fixed dose of 350 mg is motivated by clinical results demonstrating comparable efficacy to the 3 mg/kg Q2W dose [32]. This shift from a weight-based dose to the fixed dose of 350 mg corresponds to a standardization on a 116 kg patient, with a lengthening of the dosing interval from 2 to 3 weeks. It should be noted that a Japanese Phase I study showed an identical efficacy and safety profile between 250 mg Q3W and 350 mg Q3W fixed doses. Exposure to cemiplimab was increased but was considered to have no clinical impact due to the wide therapeutic window [90]. Duration of treatment was also extended, first to 48 weeks in the Phase I study with a patient who showed a durable response even after discontinuation of treatment [88] and then to 96 weeks in the Phase II study of 59 patients [89]. There are limited pharmacokinetic data available on cemiplimab and, like nivolumab and pembrolizumab, clearance appears to be time-dependent with patient weight as a covariate, although no studies have shown a significant impact. The half-life of cemiplimab was estimated to be 19.4 days. To date, no pharmaco-economic study has evaluated the impact of choosing this fixed dose over other doses.

## Therapeutic antibodies targeting PD-L1

### Atezolizumab, humanized IgG1

#### Clinical development

The Phase I PCD4989g study evaluated the efficacy and safety of atezolizumab in 277 patients with advanced/metastatic tumors. During the dose escalation period, patients received 0.01 (*n* = 1), 0.03 (*n* = 1), 0.1 (*n* = 1), 0.3 (*n*  = 3), 1 (*n* = 3), 3 (*n*  = 3), 10 (*n*  = 6) and 20 (*n* = 12) mg/kg Q3W for 16 cycles. The MTD was not reached. In the expansion phase, the highest doses of 10, 15, and 20 mg/kg were selected. No new investigations on lower doses with larger numbers of patients were carried out [37]. In the expansion period, all patients with urothelial carcinoma received 15 mg/kg Q3W. The choice of this dose was based on the demonstrated antitumor efficacy for doses ranging from 1 to 20 mg/kg and the fact that no dose-limiting toxicity was reached for all doses studied. This dose allowed for steady concentration of 6 µg/mL overtime associated with maximum occupancy of the target receptor [91]. However, preliminary study results indicate that a minimum serum concentration ≥ 6 g/mL is achieved by more than 90% of patients with the 4 mg/kg Q3W dose [92]. The dose selected for use in clinical development is therefore almost four times higher than the minimum dose that appears to be needed. The literature is quite thin on the switch to the 1200 mg fixed dose Q3W. Selection of the Q3W interval is supported by pharmacokinetic data and allows for easier combination with current chemotherapy protocols. Clinical development was pursued only with fixed dose in the non-randomized Phase II IMvigor210 trial and in subsequent studies [38, 93, 94]. In the treatment of NSCLC, small cell lung cancer, and hepatocellular carcinoma, only the fixed dose of 1200 mg Q3W was evaluated [95–103]. Doses of 840 mg Q2W, or 1680 mg Q4W for NSCLC were proposed based on modeling studies [104, 105]. In the treatment of triple negative breast cancer, the choice of 840 mg Q2W was not argued for but fits more easily into the nab-paclitaxel regimen [106].

#### Potential dose and schedule optimization for atezolizumab

Atezolizumab has a half-life of 27 days with a clearance that non-significantly decreases at steady state [107]. The pharmacokinetic profile is linear between doses ranging from 1 to 20 mg/kg. No dose-exposure relationship has been shown for efficacy or safety in the different types of cancer evaluated [37, 107]. Efficacy was comparable for doses ranging from 10 to 20 mg/kg Q3W. Preclinical and clinical data suggest that a dose of 4 mg/kg Q3W is enough to achieve 90% target receptor occupancy [92]. Atezolizumab was initially developed at a dose of 15 mg/kg. Two in silico modeling analyses showed comparable efficacy and safety for doses of 15 mg/kg Q3W, 840 mg Q2W, 1200 mg Q3W and 1680 mg/kg Q4W, corresponding to a serum concentration of 6 mg/mL [104, 105]. In clinical studies, the average patient weight was 70 to 80 kg, the switch to the fixed dose of 840 mg corresponds to the dose for a 56 kg person. The shift to the fixed dose corresponds to a decrease in dose. However, it remains well above the minimum effective dose of 4 mg/kg [92]. Based on pharmacokinetic and efficacy data, it was proposed that the fixed dose of 840 mg be administered at intervals of more than 2 weeks [108]. This is because there is a large difference between the minimum steady-state concentration for approved doses (> 100 µg/mL) and the minimum effective serum concentration (6 µg/mL) [49]. Therapeutic monitoring could allow significant reduction in the dose and/or dosing frequency, especially in responders with decreasing clearance.

### Avelumab, human IgG1

#### Clinical development

Initial clinical development with the Phase Ia JAVELIN Solid Tumor dose-escalation study evaluated four doses of avelumab (1, 3, 10 and 20 mg/kg Q2W to unacceptable progression or toxicity) in 53 patients with advanced solid tumors. The MTD was not reached. Pharmacokinetic data, favorable safety profile and promising antitumor efficacy led to the selection of the 10 mg/kg Q2W dose for clinical development [33–36]. This regimen then evolved to the 800 mg Q2W fixed dose [109].

#### Potential dose and schedule optimization for avelumab

As with other ICIs, avelumab is now recommended at the fixed dose of 800 mg Q2W based on an in silico modeling study, from data of 1827 patients, demonstrating equivalence with the weight-based dose of 10 mg/kg Q2W in these two indications [109]. This corresponds to a standardization on a 80 kg patient [34, 35]. Avelumab is the only ICI that was not designed to omit antibody-dependent cell-mediated cytotoxicity (ADCC), which was suspected to promote lysis of PD-L1 positive immune cells. The ability of avelumab to mediate ADCC of tumor cells versus peripheral immune cells was conducted in controlled in vitro experiments using NK cells as effectors from healthy donors and cancer patients and showed that avelumab can mediate ADCC of PD-L1 expressing tumor cells, but not against PD-L1 expressing immune cells [110]. Additionally it may enhance the ability to promote cancer cell killing since PD-1 has been shown to be upregulated on NK cells of patients with various tumors, and blocking the PD-1/PD-L1 interaction may increase NK cell activity against PD-L1-expressing tumor cells [111]. The in vivo impact of the ADCC phenomenon on the antitumor activity of avelumab is not clearly defined [112].

Avelumab does not have the typical pharmacokinetic profile of IgG1. Maximum concentration is achieved between the doses of 3 and 20 mg/kg and a PD-1 receptor occupancy rate of more than 90% is achieved with 3 mg/kg (evaluated in 18 patients receiving 3–20 mg/kg) [33]. The half-life of avelumab is particularly short (3.9 days for a 10 mg/kg dose and 4.1 days for a 20 mg/kg dose) and the exposure/response relationship does not plateau at the doses evaluated, suggesting that clearance is primarily related to target saturation. The currently available dosing regimen therefore appears to be optimized, which does not motivate the implementation of an alternative dosing regimen. As with other ICIs, clearance of avelumab varies over time and the magnitude of this decrease is greater in responders among patients with Merkel cell carcinoma. This change in clearance over time may be the result of improved disease status after effective treatment [49, 113]. The question remains whether a higher dose level for fast clearance patients would lead to a better response or whether maintaining long-term treatment would be sufficient.

### Durvalumab, human IgG1

#### Clinical development

Doses ranging from 0.1 to 10 mg/kg administered every 2, 3 or 4 weeks were assessed during the Phase Ib dose escalation study. In NSCLC, the doses evaluated were 3, 10, 15, 20 mg Q4W and 10 mg/kg Q2W in combination with tremelimumab. The MTD was reached for patients with NSCLC receiving 20 mg/kg of durvalumab Q4W + tremelimumab at 3 mg/kg, the toxicity was related to tremelimumab. Doses of durvalumab at 20 mg/kg Q4W in combination with tremelimumab at 1 mg/kg [39] and at 10 mg/kg Q2W in monotherapy were selected [40, 114]. This dose of 10 mg/kg Q2W is still used in NSCLC, but the dosing has evolved to a fixed dose in the treatment of small cell lung cancer with a dose of 1500 mg Q3W when combined with a chemotherapy and Q4W when used in monotherapy [41, 115].

#### Potential dose and schedule optimization for durvalumab

The half-life of durvalumab is 18 days. Pharmacokinetics are linear for doses ≥ 3 mg/kg with clearance decreasing over time, although not considered clinically relevant. interpatient variability in clearance was estimated at 27% [116]. Promising antitumor efficacy was found for doses greater than 10 mg/kg Q4W, based on small patient cohorts [39]. At the approved dose of 10 mg/kg Q2W, the minimum steady-state serum concentration is 91.9 µg/mL, which is well above the effective minimum serum concentration of 50 µg/mL [49]. In silico pharmacokinetic simulation analysis would support a switch to the 750 mg Q2W or 1500 mg Q4W fixed dose, corresponding to a standardization on a 75 kg patient from the 10 mg/kg weight-based dose. Simulated pharmacokinetic profiles show serum concentrations of durvalumab strictly above the 50 µg/mL threshold, exceeding 100 µg/mL for the 10 mg/kg dose and the two fixed doses evaluated [116]. The change in dosing interval from Q2W to Q4W appears justified and safe, and it is possible that lower doses of durvalumab or a longer dosing interval would also be effective without impacting treatment outcomes, but this has not been evaluated.

## Therapeutic monitoring of immune checkpoint targeted antibodies

Clinical development of all monoclonal ICIs was initially based on a weight-adjusted dose to reduce potential interpatient variability. Given the wide therapeutic range of these antibodies, their selective mode of action and their favorable safety profiles, the idea of using fixed versus weight-adjusted dosing quickly emerged [117, 118]. Fixed doses offer many advantages by limiting the risk of dose calculation errors, by facilitating the preparation and management of leftovers by pharmacies, and by facilitating administration for the care team, at the expense of an increase in the individual cost per patient of these treatments [2, 119]. The rationale for using fixed doses is based on in *silico* modeling studies [82, 117, 118]. A limitation of these types of studies is their inability to consider changes in clearance of ICIs during treatment and the development of anti-drug antibodies, as well as outlier patients. Confirmations through prospective clinical studies are therefore needed. Clearance is increased in patients with high tumor burden whose protein consumption is higher [67, 87], while it decreases in responders [67, 113]. Determination of clearance to provide early information on the evolution of the response could be relevant in these situations.

The economic impact of switching to fixed doses seems also significant. A recent French study estimated that the administration of a fixed dose compared to the weight-adjusted dose for nivolumab and pembrolizumab would represent an increase in the annual health system budget of €55,162,211 in 2017 [119]. In the USA, the use of weight-adjusted dosing of pembrolizumab in the NSCLC would save 24% of the total annual cost of pembrolizumab, estimated at $825,630,583, compared to the fixed dosing [120]. In addition to this over cost of treatments, the weight references of fixed dosing protocols do not correspond to the average weight of patients being treated for cancer. Indeed for pembrolizumab, the change from the 2 mg/kg dose to the fixed dosing of 200 mg Q3W corresponds to a standardization on a patient of 100 kg, whereas in common practice, the patients had an average weight of 76 kg [119, 120], and 77.2 kg during clinical development [81]. A modeling study showed that the fixed dose of 154 mg was equivalent to 2 mg/kg Q3W [81]. This reduced dose of 154 mg (compared to the approved 200 mg) would correspond to a standardization on a 77 kg patient, which is closer to the actual average weight of patients. The same is true for nivolumab, with a standardization on a 80 kg patient, while patients had an average weight of 71 kg [119].

Given the similar efficacy and safety profile between weight-adjusted and fixed dosing, pharmacokinetic data show that switching to a fixed dosing leads to overexposure to treatment for low-weight patients and underexposure for high-weight patients [117]. In order to maintain the ease of use of a fixed dose, it may be worthwhile to add flexibility to the current fixed doses by determining a lower fixed dose for low-weight patients and a higher fixed dose for overweight patients. Another option would be to reintroduce the possibility of weight-adjusted dosing to give more flexibility in dose adjustment without compromising efficacy, for the benefit of the patient and the cost to health systems. Follow-up and adaptation via therapeutic drug monitoring have never yet been tested prospectively in immuno-oncology. Patient management would be personalized and could reduce the number of hospital visits by lengthening the dosing schedules [49]. All PD-1 and PD-L1 inhibitors, except avelumab, showed a plateaued exposure–response curve at the doses evaluated in clinical studies. Those data are obtained from strictly selected patient populations. It is very likely that variations in pharmacokinetics are greater in real-life setting [6]. In addition, the decrease in clearance of therapeutic antibodies over time in responders, described particularly for nivolumab, pembrolizumab and avelumab, would indicate that administration of fixed doses at fixed dosing intervals would lead in exposure to doses well above the effective determined dose. A recent article in a small population reported the efficacy and safety of using a sixfold lower dose of nivolumab (40 mg Q2W) and twofold lower dose of pembrolizumab (100 mg Q3W) in relapsed/refractory classical Hodgkin lymphoma, as well as other retrospectively reported cases [121, 122]. The absence of an apparent relationship between exposure and efficacy/tolerance at any dose indicates the wide therapeutic window for these antibodies. Additionally, the switch to fixed dosing for most of these treatments generally leads to an increase in dosage. All these arguments highlight the need to optimize the dose of ICIs over time and depending on the pathology, to ensure that the patient receives the most appropriate treatment dose. This optimization can be done by assessing the clearance via a blood assay and then pharmacological modeling.

The implementation of therapeutic monitoring requires the identification and characterization of reliable and easily measurable parameters. The tumor response can be used, as well as the measurement of therapeutic antibodies in the blood. Other parameters such as albumin levels over time have been proposed as a surrogate for durvalumab antibody clearance [116] and could be applied to other ICIs [108]. It is important to consider other factors such as interpatient variability, antigen turnover/density, tumor burden and/or level of tumor-infiltrating lymphocytes or inflammation. These parameters could be complemented by the identification of genetic biomarkers to determine, prior to the initiation of treatment, categories of patients (responders vs. suspected non-responders) and to more specifically adapt this therapeutic monitoring [123]. All these parameters are to be defined for each ICI. The selection and characterization of the parameters for this follow-up are the next challenge.

## Conclusion and future perspectives

Overall, few treatment regimens (doses and dosing intervals) have been thoroughly evaluated in the clinical development of ICIs and, given the favorable safety profile of this family of molecules (with the exception of anti-CTLA4), dose selection has been at the higher end of the dose range, well beyond the likely minimum effective doses, along with dosing intervals and duration of treatment. For anti-CTLA-4 compounds, a concentration–response relationship has been clearly established, whereas for most anti PD-1/PD-L1 compounds, clearance appears to be an early indicator of response to treatment. Its use to adjust the dose during treatment and improve efficacy is unlikely, as the evolution of clearance may be a consequence of the pathophysiological situation [4]. Nevertheless, it seems interesting to use it to identify early non-responders. On the other hand, the use of this clearance to adjust the dosing intervals is an interesting prospect, allowing a lengthening of the dosing intervals when the clearance is decreased, to the benefit of the patient and the cost. As for the duration of treatment, ICIs function as reactivators of the antitumor immunity, which raises the question of the possibility of extending the dosing intervals once the immune response has been restored. Prospective pharmacological data are lacking to address these issues. The implementation of prospective studies and real-world evidence, both in terms of pharmacokinetics and pharmacodynamics, would enrich the data established from selected patients in development trials. The issue of using minimum effective doses as a starting point and assessing pharmacokinetic parameters after the first cycle to increase the dose when clearance is fast or lengthen the interval between doses when clearance is slow would be interesting to address. Whether in terms of clinical outcomes, ease of patient management, and cost savings, compared to standardized management as recommended today, including patients of extreme body weight.

## Data Availability

Not applicable.

## Abbreviations

ICI: Immune Checkpoint Inhibitor
CTLA-4: Cytotoxic T lymphocyte antigen-4
PD-1: Programmed cell death protein-1
IgG: Immunoglobulin of isotype G
ORR: Objective response rate
PFS: Progression-free survival
OS: Overall survival
MTD: Maximum tolerated dose
HR: Hazard ratio
ADCC: Antibody-dependent cell-mediated cytotoxicity
QxW: Every x weeks
mNCSLC: Metastatic Non-small cell lung cancer
wt: Wild type
RCC: Renal cell carcinoma
cHL: Classical Hodgkin lymphoma
UC: Urothelial cancer
HNSCC: Head and neck squamous cell carcinoma
MCC: Merkel cell carcinoma
mCRC: Metastatic Colorectal cancer
HCC: Hepatocarcinoma
GEJ: Gastroesophageal junction
PMBCL: Primary mediastinal B-cell lymphoma
SCLC: Small cell lung cancer
SCC: Squamous cell carcinoma
TNBC: Triple negative breast cancer
NMIBC: Non-muscle-invasive bladder cancer
ESCC: Esophageal squamous cell carcinoma
BCC: Basal cell carcinoma
